# Sex and Polytobacco Use among Spanish and Turkish University Students

**DOI:** 10.3390/ijerph16245038

**Published:** 2019-12-11

**Authors:** Sílvia Font-Mayolas, Mark J. M. Sullman, Maria-Eugenia Gras

**Affiliations:** 1 Quality of Life Research Institute, Universitat de Girona, 17071 Girona, Spain; eugenia.gras@udg.edu; 2 Department of Social Sciences, University of Nicosia, Nicosia 2417, Cyprus; sullman.m@unic.ac.cy

**Keywords:** electronic cigarettes, cigarettes, waterpipe, hookah, polytobacco use, young adults, sex, cognitions, attitudes

## Abstract

Polytobacco use has become increasingly popular among young adults, particularly males, and can be defined as the concurrent use of regular cigarettes and other tobacco products (e.g., e-cigarettes). The present study investigated the use of legal smoking products (cigarettes, waterpipe and electronic cigarettes) among young adults (*n* = 355) in Spain and Turkey. The survey measured demographics, lifetime and past month tobacco use, waterpipe and e-cigarette use, whether waterpipes and e-cigarettes contained nicotine and reasons for using these substances. The majority of the Turkish (men = 80% and women = 63.9%) and Spanish sample (men = 61.4% and women = 69.3%) were polytobacco users. The most common reason for using e-cigarettes was “to experiment, to see what is like” (Turkish sample: men 66.7% and women 57.1; Spanish sample: men 72.7% and women 93.8%). The most common reason to use regular cigarettes was “to relax and relieve tension” (Turkish sample: men 88.9% and women 77.6%; Spanish sample: men 78.1% and women 76%), while for waterpipe users, the most common reason was “to experiment, to see what it is like” (Turkish sample: men 93.3% and women 80%; Spanish sample: men 78.9% and women 93.8%). The implications for prevention and future research are discussed.

## 1. Introduction

Polydrug use is defined as the consumption of more than one type of drug by an individual [1]. The use of at least two different psychoactive substances among young adults is common and significantly contributes to the addiction problem [2,3,4].

The polydrug use phenomenon has also been described specifically among nicotine users, in what some authors have called “Polytobacco use” or the concurrent use of cigarettes and other tobacco products [5]. Alternative nicotine and tobacco products (ANTP) include electronic-cigarettes and waterpipes (also known as hookah, shisha or narghile), which have recently increased in popularity [6,7,8,9]. Hookah use has been identified as a predictor of the subsequent use of tobacco products, such as e-cigarettes [10]. Hookah use has also been found to be associated with lung cancer and other types of respiratory illness [11,12,13]. Furthermore, although in lower concentrations than in regular cigarettes, a number of toxic substances, including carcinogens, have been found in the vapor from electronic cigarettes [14].

The WHO Study Group on Tobacco Products Regulation identified regional patterns of waterpipe smoking with traditional use in the Eastern Mediterranean region (e.g., Turkey) and emerging use in the European region (e.g., Spain). Turkey and Spain have developed public policies to reduce cigarette use and more recently e-cigarette use. Turkey has also developed policies to reduce waterpipe use, such as the addition of warning labelling on waterpipe bowls [10], but there has been little research about waterpipe use in Spain. Investigating the current pattern of polytobacco use in those two countries could contribute to our understanding of the polytobacco use phenomenon in these two regions.

Previous research in North America has found substantial gender differences in polytobacco use, in that more males (than females) were polytobacco users and male lifetime polytobacco users were more likely to use alcohol and drugs than women [6,15]. The findings regarding waterpipe and e-cigarette use have been inconsistent, with some research finding men were more frequent users [16] and others finding no association [17,18]. Examining the different typologies of polytobacco use by sex may be a useful strategy to understand this behavior and to develop tailored interventions [19].

The main aim of the present study was to describe lifetime and current use of electronic cigarettes, regular cigarettes, waterpipes and current polytobacco use among young adults by sex and by country. The second aim was to investigate whether e-cigarettes and waterpipes were used to smoke nicotine and whether there were any sex differences. The third purpose was to examine reasons for differences in the use of e-cigarettes, regular cigarettes and waterpipes by sex and by country.

## 2. Materials and Methods

### 2.1. Participants and Procedure

A convenience sample of university students (*n* = 355) completed an online survey. Data were collected during the 2018/19 academic year. Participants were recruited using the university online platform “Moodle” in Spain and by emailing students attending a Turkish university in Northern Cyprus (Turkish).

This cross-sectional survey involved emailing a link to the students taking two compulsory courses. After clicking on the link, the students were presented with an information sheet that was followed by a page asking for their informed consent. Then students were asked to complete an online questionnaire about the prevalence of the three types of smoking behaviors. Students could complete the questionnaire in the privacy of their own rooms or homes. After completing the questionnaire, they were presented with a debriefing page and provided with details regarding how to contact the researcher, if they wanted to withdraw their data, had additional questions or wanted to know about the results. This study was approved by the Human Research Ethics Committee at Middle East Technical University—Northern Cyprus Campus.

### 2.2. Measures

#### 2.2.1. Demographics

Respondents reported their sex, age and ethnicity.

#### 2.2.2. Lifetime Use of Electronic Cigarettes, Regular Cigarettes and Waterpipe/Hookah

Participants answered the following items: “Have you ever smoked electronic cigarettes (e-cigarettes) or vaped?”, “Have you ever smoked tobacco from a waterpipe (also known as hookah, shisha, narguile)?” and “Have you ever smoked regular cigarettes?”. Response options were “yes/no”.

#### 2.2.3. Use of E-Cigarettes, Regular Cigarettes and Waterpipe/Hookah in the Past 30 Days

Students responded to the question “How frequently have you smoked e-cigarettes during the past 30 days?”. The same question was adapted to hookah and regular cigarettes. Response options were “never”, “occasionally”, “once a week”, “more than once a week, but not every day”, and “every day”.

#### 2.2.4. E-Cigarettes and Waterpipe Nicotine Contents

Participants were asked whether they had smoked e-cigarettes during the past 30 days and whether the e-cigarettes were: “with nicotine”, “without nicotine” and “with and without nicotine” [20] (Ministry of Health, Social Services and Equality, 2017). Participants that had used waterpipe/hookah during the past 30 days were also asked whether the waterpipe(s) were: “with tobacco”, “with non-tobacco or herbal shisha” and “with marijuana or hashish” [21].

#### 2.2.5. Reasons for Vaping/Smoking

Participants who reported having vaped or smoked at some time were asked “What were your main reasons for using an e-cigarette/regular cigarette/waterpipe?” [22]. Thirteen possible reasons were given: “to experiment, to see what it is like”, “because it tastes good”, “because of boredom, nothing else to do”, “to have a good time with my friends”, “to relax or relieve tension”, “because it looks cool”, “to help me quit regular cigarettes”, “to get high”, “because I am “hooked”—I have to have it”, “because friends or family member used them”, “because e-cigarettes/waterpipe without nicotine are less harmful than regular cigarettes”, “because e-cigarettes/waterpipe with nicotine are less harmful than regular cigarettes”, and ”because friends or family members permitted e-cigarettes/waterpipes, more than regular cigarettes” [22,23]. Students were asked to evaluate each of the 13 reasons using the following response options: “definitely yes”, “probably yes”, “probably no” and “definitely no”.

### 2.3. Analysis

All the analyses were conducted by sex and subsample. Chi-square tests and the contingence coefficient of effect size were applied to analyze lifetime and current substance use. To meet the assumptions of the chi-squared test, the categories “occasionally” and “once a week” and “more than once a week” and “every day” were combined. Chi-square tests were also used to study e-cigarette contents and to analyze reasons for vaping/smoking. The data analysis categories “definitely yes” and “probably yes” were combined and considered to be an “important reason”. Further, the categories “definitely no” and “probably no” were combined and were not considered to be an “important reason”. Fisher’s exact test was used when expected frequencies were lower than 5. All the analyses were performed using SPSS Version 23 (IBM, Armonk, NY, USA).

## 3. Results

The Spanish sample was composed of 236 participants (75.4% female, mean age: 20.7 years (SD = 1.6), ethnicity: 93.6% European, 1.3% Asian, 0.4% Black, 0.4 Turkish, 4.2% Other). The Turkish sample was composed of 119 participants (68.5% female, mean age: 22.5 years (SD = 1.4), ethnicity: 95.5% Turkish, 1.8% Asian, 2.7 Other). The response rates were 90.76% in the Spanish sample and 98.34% in the Turkish sample.

### 3.1. Lifetime Prevalence Use

Table 1 shows the lifetime prevalence of having used e-cigarettes, regular cigarettes and waterpipes. The prevalence in Turkey was higher than in Spain, for both men and women, but these differences were only statistically significant for waterpipe consumption among men. Moreover, in both subsamples (Spanish: (X^2^_(1)_ = 6.2; *p* = 0.01; Φ = 0.16; Turkey: X^2^_(1)_ = 5.7; *p* = 0.02; Φ = 0.22) significantly more men than women had used e-cigarettes. The consumption of regular cigarettes and the use of waterpipes were similar for women and in men in both samples (Cigarettes: Spain: X^2^_(2)_ = 0.8; *p* = 0.77; Turkish: X^2^_(1)_ = 1.5; *p* = 0.22. Waterpipe: Spain: X^2^_(2)_ = 0.1; *p* = 0.73; Turkish: X^2^_(1)_ = 2.3; *p* = 0.13).

There was only one male in the Turkish sample who only consumed electronic cigarettes and the consumption of electronic cigarettes with regular cigarettes or with waterpipes was also rare. The consumption of regular cigarettes with waterpipes (i.e., polytobacco use) was much more common, ranging from 19% to 36% of the participants, depending on their sex and country of origin (Table 2). Polytobacco use with e-cigarettes, regular cigarettes and waterpipes was more common among men, particularly Turkish men. Participants were placed into three groups: those who did not use any of the three substances, those who only used one, and those who used two or all three. A chi-square test was performed to compare the polytobacco use of the Spanish and Turkish men (X^2^_(2)_ = 4.3; *p* = 0.12) and women (X^2^_(2)_ = 2.8; *p* = 0.25), but no significant differences were found. When we compared men and women in each country, there were no significant differences in the Spanish sample (X^2^_(2)_ = 1.4; *p* = 0.50) or in the Turkish sample (X^2^_(2)_ = 3.1; *p* = 0.22).

In total, 61.4% of Spanish men, 80% of Turkish men, 69.3% of Spanish women and 63.9% of Turkish women were classified as polytobacco users, but there were no significant differences by country (Men: X^2^_(1)_ = 2.9; *p* = 0.09; Women: X^2^_(1)_ = 0.5; *p* = 0.46) or sex (Spanish: X^2^_(1)_ = 0.9; *p* = 0.33; Turkish: X^2^_(1)_ = 2.4; *p* = 0.12).

### 3.2. Use in the Past 30 Days

The frequency of e-cigarette consumption over the past 30 days was similar in the Turkish sample and in the Spanish sample, both in men and women (See Table 3). The proportion of daily consumers was very low, particularly in women. If we compare the consumption of men and women in the two sub-groups, there were no significant differences in the Spanish sample (X^2^_(2)_ = 3.7; *p* = 0.15) or the Turkish sample (X^2^_(2)_ = 3.1; *p* = 0.21).

The frequency of regular cigarette consumption over the past 30 days was also higher in the Turkish sample, with more than 50% of Turkish men and more than one-third of Turkish women reporting that they consumed cigarettes on a daily basis. In the Spanish sample, the percentages were much lower and significant differences were found with the Turkish sample for both sexes (Table 4). If we compare the consumption of men and women within each country, there were no significant differences in the STpanish sample (X^2^_(2)_ = 0.9; *p* = 0.62) or the Turkish sample (X^2^_(2)_ = 1.7; *p* = 0.43).

The frequency of waterpipe consumption over the past 30 days was higher in the Turkish sample, with more than 50% of Turkish men reporting using a waterpipe every day. The differences between the two countries were statistically significant for both men and women (Table 5). If we compare the consumption of men and women within the two countries, there were no significant differences in the Spanish sample (X^2^_(2)_ = 3.1; *p* = 0.21; Φ = 0.12), but in the Turkish sample significantly more men regularly use a waterpipe (X^2^_(2)_ = 6.1; *p* = 0.048; Φ = 0.23).

### 3.3. E-Cigarettes and Waterpipe Nicotine Content

Table 6 shows the percentage of e-cigarette users, according to the nicotine content. Men in both populations reported similar frequencies of nicotine in e-cigarettes, but Turkish women use e-cigarettes with nicotine more frequently than Spanish women and Spanish women use with and without nicotine more frequently.

Table 7 shows the percentage of waterpipe users according to the additive. Only data from the Spanish sample is available for this variable, due to a problem with the data collection. The most frequent option was to use a waterpipe with herbal shisha, but many men only use it with tobacco.

### 3.4. Reasons for Vaping/Smoking

Table 8 shows the percentage of participants that answered “definitely” or “probably” to each option as an important reason for using e-cigarettes. In both countries, the reason men most frequently considered important was “to experiment—to see what it is like”. This reason was also identified as very important for more than 90% of Spanish women. In contrast, the most frequently selected reason among Turkish women was “because it tastes good”. In general, the percentage of participants who reported each option as an important reason to use electronic cigarettes did not differ significantly between the two countries, except in the case of “to relax or relieve tension”, with more Turkish men, than Spanish men (X^2^_(1)_ = 5.1; *p* = 0.02; Φ = 0.38), reporting this to be an important reason (see Table 8). Moreover, there were no significant differences in the percentage of men and women who considered each reason to be important in either country.

The item participants reported as being the most important reason to use regular cigarettes was “to relax and relieve tension” (Table 9). There were also other reasons that were considered important by more than 50% of the sample, which were: “to experiment, to see what it is like” and “have a good time with friends”. Many Turkish men also reported that they used regular cigarettes “because of boredom”, which was significantly higher than for Spanish men. Significantly more Turkish women, than Spanish women, reported boredom as an important reason to use regular cigarettes. In addition, more Turkish men, than Spanish men, also reported that an important reason was “because friends or family members used them”. Men and women did not differ in their reasons for using regular cigarettes, with two exceptions: more Turkish men than Turkish women reported that they use regular cigarettes “because it looks cool” (X^2^_(1)_ = 4.7; *p* = 0.03; Φ = 0.25) and “because they are hooked” (X^2^_(1)_) = 6.7; *p* = 0.01; Φ = 0.30).

The three most important reasons to use waterpipes, as reported by the participants, were: “to experiment, to see what it is like”, “have a good time with friends” and “because it tastes good”. More Spanish women reported that “to experiment, to see what it is like” and to “have a good time with Friends” were important reasons to use a waterpipe, but more Turkish women reported their important reasons as “to help themselves quit regular cigarettes”, “because they are hooked” and “because waterpipes with nicotine are less harmful than regular cigarettes” (see Table 10). More Turkish men reported that “because friends and family members used them” as an important reason for using waterpipes, than did Spanish men. Spanish men also differed significantly from Spanish women across three reasons. More Spanish women rated the following two items to be important “to experiment, to see what it is like” (X^2^_(1)_ = 6.9; *p* = 0.01; Φ = 0.22) and “because it tastes good” (X^2^_(1)_ = 12.1; *p* = 0.001; Φ = 0.29), while more Spanish men reported an important reason to be “because they are hooked” (X^2^_(1)_ = 4.2; *p* = 0.04; Φ = 0.18). In the Turkish sample, men and women only differed significantly in one reason for using a waterpipe, with more men answering “because it looks cool” (X^2^_(1)_ = 7.8; *p* = 0.001; Φ = 0.32).

Finally, the outstanding results of the present research were collected and presented in Table 11.

## 4. Discussion

The first purpose of the present study was to investigate the lifetime and current use of electronic cigarettes, regular cigarettes, waterpipes and current polytobacco use among young adults, by sex and country. The data showed that lifetime e-cigarette use was higher in the Turkish sample, than in the Spanish sample. Sex differences were also found in the two samples, with more men having used e-cigarettes in their lives. Therefore, it appears that women who try e-cigarettes are more likely to become regular users than men, among the young adults in these samples.

Since 2013, electronic cigarette use has been regulated by law in Turkey and their sales are prohibited to anyone under 18 years of age [24]. Surprisingly, there was no data available about electronic cigarette use in Turkey from the Global Data Tobacco Survey [25] and, to our knowledge, no other data about e-cigarette use in Turkey has been published. The level of e-cigarette use found here among young Turkish adults shows the need for more large-scale prevalence studies in this country to investigate the use of this alternative nicotine product.

The Global Data Tobacco Survey reported the use of electronic cigarettes among the general population in Spain to be 2% (2% of men and 1.9% of women) [25]. Furthermore, the most recent national survey of Spain showed that the prevalence figures for e-cigarette use in young adults were: lifetime—15.1% of men and 10.9% of women; past 30 days—4.3% of men and 2.4% of women; and daily—1.5% of men and 1.2% of women [26]. Higher percentages were found in the present study, providing some evidence that e-cigarette use has increased in Spain and that more preventive actions must be undertaken to alert users and potential users about health risks.

In our study, lifetime regular cigarette use appeared to be higher in men than in women, in the Turkish sample, and higher in women than in men in the Spanish sample, although these differences were not statistically significant. Daily cigarette use also appeared to differ between the two countries, with more Turkish men and more Spanish women using cigarettes on a daily basis, but these differences were not statistically significant.

In line with our research findings, the WHO Global Adult Tobacco Survey showed that regular cigarette use (daily and occasional) in Turkey was more common among young men (33%) than in young women (7.4%) [27]. This sex difference was also reported in the Turkey Statistical Health Survey, where the percentage of young male daily smokers (31.4%) was significantly higher than the percentage of young females (5.7%) [28]. Anti-tobacco policies have been implemented in Turkey over the last 10 years, including tax increases and regulations, which have affected the long-term demand for cigarettes, presumably due to price sensitivity [29]. However, more preventive measures are necessary in Turkey, in particular among young men, to reduce smoking-related diseases and to reduce the rate of smoking initiation.

The most recent national Spanish survey showed that daily cigarette use in young adults was 28.5% in men and 23.2% in women, which was higher than in previous national surveys and also higher than in our study [26]. A previous study among Spanish university students also found similar daily cigarette use in females (16.9%), as in the present research, and 17.8% among males [30]. These results provide some evidence that, despite the substantial preventive efforts undertaken by the Spanish government, cigarette smoking may again be increasing among young adults. Therefore, more campaigns are needed to reduce this addictive behavior and to promote healthy lifestyles in Spain. Furthermore, interventions targeted at university students should also help them to deal with common student problems, such as stress and boredom, without the use of nicotine.

The present study found that more than 50% of the participants had used a waterpipe at some point in their life, with no sex differences. During the past 30 days, waterpipe use was more common in Turkey than in Spain, with more male users than women in Turkey, but no sex differences were found in Spain.

Previous studies in Turkey have found current waterpipe use (daily and occasional) to be more common among young men (2.5%) than in young women (1%) and that waterpipe use has declined among the general population from 2008 (men: 4%, women 0.7%) to 2012 (men: 1.1%, women: 0.5%), possibly resulting from prevention efforts such as health warnings on waterpipes [27,31]. However, these same studies also reported evidence that waterpipe smoking was increasing globally, particularly among young adults. This finding again highlights the need to continue with prevention efforts in order to maintain the decline in waterpipe use.

There is a paucity of research about waterpipe use in Spain. One study found that approximately one-third of Spanish high school students had used a waterpipe at some point in their lives, with the proportion being higher in women than in men [32]. Furthermore, another recent study reported that 13% of Spanish high school students currently (monthly or weekly) used a waterpipe [33]. These high percentages are in accordance with the present study using young adults, which demonstrates the need to include items about waterpipe use in Spanish national surveys, in order to have more complete data. Moreover, in line with the most recent National Spanish Drug Plan more preventive materials should be created to alert people about this new way of socializing in Spain and its health consequences [9].

One of the main aims of this study was to identify polytobacco use by sex and country among tobacco users. In the Turkish sample, 80% of men and 63.9% of women were polytobacco users, while in the Spanish sample, 61.4% of men and 69.3% of women were polytobacco users, although these differences (country or sex) were not statistically significant. In the same line, with previous research on Polish adolescents, dual use (regular cigarette + e-cigarette) increased over time, from 4% in 2010–2011 to 23% in 2013–2014, while exclusive regular cigarette use declined over this time (from 21% to 15%) [34]. Furthermore, research about polytobacco use among North American adolescents found that 81% of e-cigarette users also used at least one other tobacco product [35]. In a similar study among North American adolescents and young adults, 55.9% were classified as polytobacco users [36]. The results of the present study are also consistent with previous research among North American young adults that reported using at least two tobacco products, with shisha being the most common, followed by regular cigarettes and e-cigarettes [37]. The tendency toward polytobacco use is worrying, since young adults who use more tobacco products are at a greater risk for increased regular cigarette smoking and also for maintaining this polytobacco pattern of use [38].

The second purpose of the present study was to find out whether e-cigarettes and waterpipes were used with nicotine and whether there were sex and country differences. Within e-cigarettes smokers, in the Turkish sample, 56.3% of men and 57.9% of women answered that their e-cigarettes contained nicotine, while in the Spanish sample, these proportions were 31.3% of men and 11.5% of women. Our Spanish data were lower than those reported in the last National Spanish Drug survey, which found 48.4% of young adult men and 41% of young adult women smoked e-cigarettes with nicotine [26]. However, our research found that a higher proportion of the participants reported that they sometimes include nicotine in their e-cigarettes (37.5% men and 61.5% women), than was found in the most recent National Spanish Drug survey (11.5% men and 17.2% women).

Within waterpipe users, in the Spanish sample, the most frequent answer among males was that they smoked waterpipes with tobacco (36.1%), while among women the most frequent answer (57.1%) was that they smoked waterpipes with non-tobacco or herbal shisha. Approximately one-third part of men (30.6%) and women (35.3%) smoked waterpipes, combining the following: tobacco, non-tobacco or herbal shisha or marijuana/hashish. In another study among North American students, the majority of waterpipe users (90%) reported smoking tobacco, 45% marijuana, 37% herbal shisha (non-tobacco) and 18% hashish [21]. Moreover, the present research confirms that waterpipe use is a common alternative method for using nicotine. The results of the present study are concerning, since nicotine use was common, but also because of the toxic substances that have been found in waterpipe smoke, including herbal shisha. This research demonstrates the need to disseminate information about the health risks of waterpipe use among young adults [21].

The third purpose of the present study was to examine the reasons why young adults use e-cigarettes, regular cigarettes and waterpipe and whether there were any sex or country differences. The most important reason to use e-cigarettes was “to experiment, to see what is like” (Turkish sample: men 66.7% and women 57.1; Spanish sample: men 72.7% and women 93.8%), although the most important reason for Turkish women (81.3%) was “because it tastes good”. No significant differences were found by sex in the reasons reported. The only exception was for the reason “Because it relieves tension”, which was reported more commonly by Turkish men than Spanish men. This finding among Turkish men is in line with previous studies among North American young adults, where affect regulation was the most consistent predictor of e-cigarette use [17]. The results of our research are also in agreement with previous research about the reasons for vaping or e-cigarette use among North American adolescents. The most important reasons reported were: to experiment and because it tastes good [22], and because they are available in tasty flavors, such as mint, candy, fruit or chocolate [23].

The most commonly reported reason to consume regular cigarettes was “to relax and relieve tension” (Turkish sample: men 88.9% and women: 77.6%; Spanish sample: men 78.1% and women 76%). More than 50% of the two samples also considered the following reasons to be important: “to experiment, to see what it is like” and to “have a good time with friends”. More Turkish men, than Spanish men, and more Turkish women than Spanish women gave the reason “because of boredom”. More Turkish men, than Spanish men, gave the reason “because friends or family members used them”. No significant differences were found in the reasons provided by sex, with the exception of “Because it looks cool” and “because I am hooked”, which were reported more by Turkish men than Turkish women. Our findings are also consistent with previous research among North American students, where high levels of regular cigarette use were associated with higher boredom relief and affect regulation motive scores [39].

The most important reasons to use waterpipes were “to experiment, to see what it is like” (Turkish sample: men 93.3% and women 80%; Spanish sample: men 78.9% and women 93.8%), “to have a good time with friends” (Turkish sample: men 85.7% and women 80.4%; Spanish sample: men 91.9% and women 93.8%) and “because it tastes good” (Turkish sample: men 89.3% and women 84.6%; Spanish sample: men 70.3% and women 92.6%). For Spanish women, the reasons “to experiment, to see what is like” and to “have a good time with friends” were more important than for Turkish women. Turkish women reported the following reasons to be more important than for Spanish women: “to help quit regular cigarettes”, “because I am hooked” and “because waterpipes without nicotine are less harmful than regular cigarettes”. More Turkish than Spanish men reported using waterpipes “because friends and family members used them”. In the Spanish sample, men reported “because I am hooked” more often than Spanish women, but more women answered “to experiment, to see what it is like” and “because it tastes good”. In the Turkish sample, more men than women reported the reason “because it looks cool”. The pattern of findings in the present study are in line with the findings from a study about waterpipe use among North American adolescents, which found that adolescents strongly endorsed the following statements: if my best friend offered me a hookah, I would smoke; hookah helps young people feel more comfortable; hookah helps people relieve stress; it would be easy to quit using hookah [40]. The higher perceived social acceptability of waterpipe use among friends was also found to be related to the higher odds of having ever tried a waterpipe in North American adolescents [41]. These positive cognitions about waterpipe use, compared with regular cigarettes use, could help to explain the increased use of waterpipes in recent years. In addition, a general pattern among young adults to search for alternative ways of using tobacco has been proposed to explain waterpipe use [42].

There are limitations to this study. Firstly, this cross-sectional survey does not allow any temporal conclusions about tobacco use and the reasons examined to use these substances. This work was based on a convenience sample of university students who completed an online survey, as has been the case in previous polytobacco research in North America [17]. However, there are several possible sample and method limitations, which means the data collected here does not supersede those obtained in national surveys. Furthermore, all measures relied on self-reported behavior and may therefore be biased in some way. In particular, it is likely that the “because I am hooked” reason for smoking, one of the three measured products, has been under-reported by the participants, since being addicted to something is socially undesirable. Due to the multiple statistic tests undertaken regarding the main reasons for using regular cigarettes, e-cigarettes and waterpipes, some significant results could have been found by chance. However, the present study has to be considered a first step, so that future larger studies will be conducted which are able to analyze in depth polytobacco characteristics by country and sex.

## 5. Conclusions

Although there were very few differences by sex or country, the high level of polytobacco use among young adults reported in both countries (European region vs. Eastern Mediterranean region) highlights the need to develop more integrated prevention strategies. These strategies should not only include regular cigarette use, as has usually been the case, or by separating tobacco products, but must include all alternative nicotine products, such as e-cigarettes and waterpipes. This would be the way to offer a global health risk approach to nicotine use among young adults, regardless of the form of administration. Nevertheless, it is also important that the observed differences in the reasons to use these alternative tobacco products are also taken into account. Further research is also needed to examine polytobacco use in these two countries in order to better understand these new nicotine use patterns. It is suggested that future research continues to collect data about nicotine contents for waterpipes and e-cigarettes to help policy makers and to develop more accurate prevention campaigns.

## Figures and Tables

**Table 1:** Prevalence of e-cigarette, regular cigarette and waterpipe use by country and sex, chi-square test and contingency coefficient (effect size).

Type	Male		X^2^_(1)_ (*p*)	Φ	Female		X^2^_(1)_ (*p*)
	Spain	Turkey			Spain	Turkey	
E-cigarettes	27.6%	42.9%	2.3 (0.13)	-	13.5%	21.1%	2.3 (0.13)
Cigarettes	55.2%	71.4%	2.4 (0.12)	-	57.3%	59.2%	0.08 (0.78)
Waterpipe	60.3%	80.0%	3.9 (0.05)	0.20	62.9%	65.8%	0.19 (0.66)

**Table 2:** Polytobacco use by substance and sex (%).

Polytobacco Type	Male		Female	
	Spain	Turkey	Spain	Turkey
No e-cigarettes, regular cigarettes or waterpipe use	24.1%	14.3%	28.7%	19.7%
Only e-cigarette use	0%	2.9%	0%	0%
Only regular cigarette use	12.1%	2.9%	8.4%	13.2%
Only waterpipe use	17.2%	11.4%	13.5%	15.8%
E-cigarettes + regular cigarettes	3.4%	0%	0%	1.3%
E-cigarettes + waterpipe	3.4%	0%	0.6%	5.3%
Waterpipe + regular cigarettes	19%	28.6%	36%	30.3%
E-cigarettes + regular cigarettes + waterpipe	20.7%	40%	12.9%	14.5%

**Table 3:** Frequency of e-cigarette consumption in the past 30 days by country and sex, chi-square ^1^ test and contingency coefficient (effect size).

Frequency	Male		Female	
	Spain	Turkey	Spain	Turkey
Never	84.5%	68.6%	90.4%	81.6%
Occasionally	8.6%	14.3%	6.2%	10.5%
Once a week	1.7%	5.7%	2.2%	3.9%
More than once a week	1.7%	5.7%	1.1%	1.3%
Every day	3.4%	5.7%	0%	2.6%
X^2^_(2)_ (*p*)	3.3 (0.19)		4.5 (0.10)	

^1^ To meet the assumptions of the chi-squared test, the categories “occasionally /once a week” and “more than once a week/every day” were combined.

**Table 4:** Regular cigarette consumption over the past 30 days by country and sex, chi-square ^1^ test and contingency coefficient (effect size).

Frequency	Male		Female	
	Spain	Turkey	Spain	Turkey
Never	58.6%	31.4%	57.95%	43.4%
Occasionally	15.5%	8.6%	10.7%	7.9%
Once a week	3.4%	5.7%	3.9%	1.3%
More than once a week	10.3%	2.9%	7.9%	10.5%
Every day	12.1%	51.4%	19.7%	36.8%
X^2^_(2)_ (*p*)	10.1 (0.007)		9.5 (0.009)	
Φ	0.33		0.19	

^1^ To meet the assumptions of the chi-squared test, the categories “occasionally /once a week” and “more than once a week/every day” were combined.

**Table 5:** Waterpipe consumption over the past 30 days by country and sex, chi-square ^1^ test and contingency coefficient (effect size).

Frequency	Male		Female	
	Spain	Turkey	Spain	Turkey
Never	77.6%	48.6%	82.6%	60.5%
Occasionally	15.5%	40.0%	15.2%	36.8%
Once a week	3.4%	0%	1.7%	1.3%
More than once a week	3.4%	8.6%	0.6%	1.3%
Every day	0%	2.9%	0%	0%
X^2^_(2)_ (*p*)	8.5 (0.01)		14.2 (0.001)	
Φ	0.30		0.24	

^1^ To meet the assumptions of the chi-squared test, the categories “occasionally/once a week” and “more than once a week/every day” were combined.

**Table 6:** Percentage of e-cigarette users according to the nicotine content.

Nicotine Content	Male		Female	
	Spain	Turkey	Spain	Turkey
Without nicotine	31.3%	12.5%	26.9%	26.3%
With and without nicotine	37.5%	31.3%	61.5%	15.8%
With nicotine	31.3%	56.3%	11.5%	57.9%
X^2^_(2)_ (*p*)	2.5 (0.28)		13.0 (0.001)	
Φ	-		0.54	

**Table 7:** Percentage of waterpipe users according to content.

Waterpipe Content	Male	Female
With tobacco	36.1%	12.1%
With non-tobacco or herbal shisha	37.5%	51.7%
With marijuana or hashish	0%	0.9%
With tobacco and with non-tobacco or herbal shisha	5.6%	15.5%
With tobacco and with marijuana or hashish	5.6%	2.6%
With non-tobacco or herbal shisha and with marijuana or hashish	8.3%	4.3%
With tobacco, with non-tobacco or herbal shisha or with marijuana or hashish	11.1%	12.9%

**Table 8:** Most important reasons for using e-cigarettes by country and sex, chi-square * test and contingency coefficient (effect size).

Reason	Male		X^2^_(1)_ (*p*)	Φ	Female		X^2^_(1)_ (*p*)	Φ
	Spain	Turkey			Spain	Turkey		
To experiment—to see what it is like	72.7%	66.7%	* (1)	-	93.8%	57.1%	* (0.07)	-
Because it tastes good	53.6%	60.0%	0.1 (0.83)	-	65.4%	81.3%	* (0.31)	-
Because of boredom, nothing else to do	22.2%	22.2%	* (1)	-	21.6%	33.3%	* (0.51)	-
To have a good time with my friends	31.6%	27.8%	0.1 (0.80)	-	31.4%	31.3%	0 (0.99)	-
To relax or relieve tension	20.0%	56.3%	5.1 (0.02)	0.38	16.1%	37.5%	* (0.15)	-
Because it looks cool	5.0%	22.2%	* (0.17)	-	5.4%	12.5%	* (0.58)	-
To help me quit regular cigarettes	13.3%	27.3%	* (0.35)	-	14.3%	7.1%	* (0.44)	-
To get high	0%	11.8%	* (0.49)	-	0%	0%	-	-
Because I am “hooked”—I have to have it	5.9%	22.2%	* (0.19)	-	2.8%	0%	* (0.69)	-
Because friends or family members used them	22.2%	26.7%	* (0.54)	-	17.6%	20.0%	* (0.57)	-
Because e-cigarettes without nicotine are less harmful than regular cigarettes	25.0%	35.7%	* (0.68)	-	25.0%	29.4%	* (0.75)	-
Because friends or family member allowed e-cigarettes more than regular cigarettes	0%	17.6%	* (0.10)	-	5.7%	22.2%	* (0.09)	-

* Fisher’s exact test was used when the expected frequencies were lower than 5.

**Table 9:** Most important reasons to use regular cigarettes by subsample and sex, chi-square * test and contingency coefficient (effect size).

Reason	Male		X^2^_(1)_ (*p*)	Φ	Female		X^2^_(1)_ (*p*)	Φ
	Spain	Turkey			Spain	Turkey		
To experiment—to see what it is like	58.1%	66.7%	0.4 (0.50)	-	68.6%	61.2%	0.8 (0.37)	-
Because it tastes good	25.8%	37.0%	0.9 (0.36)	-	40.4%	37.7%	0.5 (0.50)	-
Because of boredom, nothing else to do	41.4%	74.1%	6.1 (0.01)	0.33	21.6%	33.3%	5.2 (0.02)	0.19
To have a good time with my friends	51.6%	66.7%	1.3 (0.25)	-	57.3%	69.4%	2.1 (0.15)	-
To relax or relieve tension	78.1%	88.9%	* (0.23)	-	76.0%	77.6%	0.1 (0.83)	-
Because it looks cool	31.0%	33.3%	0.03 (0.85)	-	17.6%	12.5%	0.6 (0.42)	-
To get high	14.3%	20.0%	* (0.43)	-	9.9%	20.8%	3.3 (0.07)	-
Because I am “hooked”—I have to have it	28.6%	50.0%	2.6 (0.11)	-	35.3%	20.8%	3.2 (0.07)	-
Because friends or family members used them	20.0%	46.2%	4.4 (0.04)	0.28	31.0%	38.8%	0.9 (0.35)	-

* Fisher’s exact test was used when the expected frequencies were lower than 5.

**Table 10:** Most important reasons to use a waterpipe (probably or definitely yes) by country and sex, chi-square * test and contingency coefficient (effect size).

Reason	Male		X^2^_(1)_ (*p*)	Φ	Female		X^2^_(1)_ (*p*)	Φ
	Spain	Turkey			Spain	Turkey		
To experiment—to see what it is like	78.9%	93.3%	* (0.17)	-	93.8%	80.0%	6.9 (0.01)	0.21
Because it tastes good	70.3%	89.3%	3.4 (0.07)	-	92.6%	84.6%	2.5 (0.12)	-
Because of boredom, nothing else to do	45.7%	53.6%	0.4 (0.54)	-	31.2%	39.2%	1.0 (0.32)	-
To have a good time with my friends	91.9%	85.7%	* (0.34)	-	93.8%	80.4%	6.8 (0.01)	0.20
To relax or relieve tension	33.3%	50.0%	1.8 (0.18)	-	32.1%	45.1%	2.5 (0.11)	-
Because it looks cool	20.0%	39.3%	2.8 (0.09)	-	15.2%	12.0%	0.3 (0.59)	-
To help me quit regular cigarettes	0%	11.1%	* (0.08)	-	2.9%	14.3%	* (0.01)	0.21
To get high	23.5%	22.2%	0.02 (0.90)	-	9.7%	14.3%	0.7 (0.40)	-
Because I am “hooked”—I have to have it	0%	7.4%	* (0.19)	-	1.0%	12.0%	* (0.01)	0.25
Because friends or family members used them	25.7%	55.6%	5.7 (0.02)	0.30	25.7%	36.0%	1.7 (0.19)	-
Because waterpipes without nicotine are less harmful than regular cigarettes	11.4%	21.4%	1.2 (0.28)	-	9.9%	22.0%	4.1 (0.04)	0.17
Because friends or family member allowed waterpipe use more than regular cigarettes	8.8%	12.0%	0.2 (0.69)	-	9.2%	19.1%	2.9 (0.10)	-

* Fisher’s exact test was used when the expected frequencies were lower than 5.

**Table 11:** Outstanding results.

1. Lifetime prevalence of e-cigarette, cigarette and waterpipe use was high in both samples.
2. Lifetime prevalence of waterpipe use was higher in Turkish men, than in Spanish men. In women, the prevalence was similar.
3. The use of e-cigarettes was almost always linked to regular cigarette and waterpipe use.
4. Daily consumption of waterpipe and regular cigarettes was higher in the Turkish sample, than in Spanish sample, but there were no differences for e-cigarette consumption.
5. More than 50% of the Turkish participants (men and women) always use waterpipes with nicotine.
6. The most important reason to use e-cigarettes in the Spanish sample (men and women) and for Turkish men was to experiment, to see what it is like. For Turkish women, the most important reason was because it tastes good.
7. The most important reason to use cigarettes, in all cases, was to relax or relieve tension.
8. The most common reasons to use waterpipes was to have a goodtime with friends, to experiment to see what it is like and because it tastes good.
